# General anesthetic management in two patients with an anaphylaxis history cholinergic urticaria

**DOI:** 10.1186/s40981-023-00640-w

**Published:** 2023-08-02

**Authors:** Atsushi Kotera

**Affiliations:** Department of Anesthesiology, Kumamoto City Hospital, 4-1-60, Higashimachi, Higashi-Ku, Kumamoto City, Kumamoto, 862-8505 Japan

**Keywords:** Cholinergic urticaria, General anesthesia, Body temperature

## Abstract

**Background:**

Cholinergic urticaria is triggered by an increased body temperature after exercise, passive warming, or emotional stresses. The anesthetic management used for two patients with an anaphylaxis history after cholinergic urticaria is described.

**Case presentation:**

Patient 1: A 34-year-old female was scheduled for a right-side thyroidectomy. At 27 years old, she experienced post-exercise anaphylaxis and repeated episodes of hives following exercise, sunbathing, mental stresses, and the consumption of spicy foods. Patient 2: A 35-year-old female was scheduled for a laparoscopic ovarian cystectomy. At 19 years old, she experienced anaphylaxis after a long bath and then hives after hot showers, bicycle riding, and long walks. For both patients, intraoperative passive warming was not performed to prevent excessive warming, and prophylactic antiemetics and multimodal analgesia were used to minimize their perioperative stresses.

**Conclusion:**

Careful anesthetic management is necessary to prevent anaphylaxis during anesthesia in a patient with a history of cholinergic urticaria.

## Background

Cholinergic urticaria, which is characterized by pruritic pinpoint-sized wheals, is triggered by an increased body temperature with sweating after exercise, high environmental temperature, or mental stresses [1], and it can sometimes induce anaphylaxis [2]. The reported incidence of cholinergic urticaria is 0.025% in Korea, 0.7% in Thailand, 4.2% in a young Indian population (18–22 years old), and 11.2% in a young German population (15–35 years old) [3–6]. The prevalence of cholinergic urticaria has been the highest in individuals aged 20–29 years, with a male predominance, but in patients aged ≥ 40 years, its prevalence was reported to be higher in females than males [3].

Cholinergic urticaria is thought to develop following an immediate-type allergy to autologous sweat antigens or following IgE which increases in response to antigens or certain elements contained in sweat [7]. Acetylcholine injected intradermally can induce both sweating and hives, as seen in cholinergic urticaria. It is thus suggested that cholinergic urticaria is provoked by a stimulus mediated by acetylcholine [1].

In a patient with cholinergic urticaria, anesthesiologists are required to manage patients without inducing anaphylaxis during surgery. I present the anesthetic management for two patients with a history of anaphylaxis following cholinergic urticaria.

## Case presentation

### Patient 1

A 34-year-old female (height, 164 cm; weight, 45 kg) was scheduled for a right-side thyroidectomy. She had a history of allergic rhinitis. At 27 years old, anaphylaxis (dyspnea, hives on the body trunk, and loss of consciousness) after exercise in a bathroom had occurred. She was then diagnosed as having cholinergic urticaria based on her history of repeated hives following exercise, sunbathing, mental stresses, and the consumption of spicy foods. Her daily medications were loratadine 20 mg and bilastine 20 mg, and she sometimes used prednisolone 5 mg or d-chlorpheniramine maleate 4 mg as a rescue. Her preoperative white blood cell count was 4900/mm^3^, and the portion of eosinophils was 10%.

The patient’s daily medications were taken on the operative day, and her preoperative axillary body temperature was 36.7 ℃. General anesthesia was induced with 90 mg propofol, 0.25 μg/kg/min remifentanil, 200 μg fentanyl, and 40 mg rocuronium. General anesthesia was maintained with 5.0 mg/kg/h propofol and 0.2–0.3 μg/kg/min remifentanil. For antiemesis, 6.6 mg dexamethasone and 4 mg ondansetron were given, and 350 μg fentanyl, 800 mg acetaminophen, and 50 mg flurbiprofen were administered for multimodal analgesia.

The patient’s intraoperative body temperature was continuously measured by a rectal thermometer, and it ranged from 37.2° to 37.4 ℃. The surgical data were as follows: surgical duration, 95 min; anesthesia time, 131 min; blood loss, 21 g; urine output, a small amount, total infusion volume, 900 mL. The postoperative axillary body temperature measured periodically during the patient’s hospitalization ranged from 36.5° to 36.9 ℃, and perioperative cholinergic urticaria was not observed.

### Patient 2

A 35-year-old female (height, 153 cm; weight, 50 kg) was scheduled for a laparoscopic ovarian cystectomy. She had a history of atopic dermatitis and cold-induced urticaria. At 19 years old, anaphylaxis (vomiting, coughing, hives on the body trunk, and edema of lips and eye lids) after long-time bathing had occurred. She was then diagnosed with cholinergic urticaria based on her history of repeated hives following hot showers, bicycle riding, and long walks. Her daily medications were levocetirizine 5 mg, cimetidine 400 mg, d-chlorpheniramine maleate 6 mg, and suplatast tosilate 200 mg. Her preoperative white blood cell count was 5000/mm^3^, and the portion of eosinophils was 4.8%.

The patient’s daily medications were taken on the operative day, and her preoperative axillary body temperature was 36.5 ℃. General anesthesia was induced with 7.0 μg/mL propofol (target controlled infusion; TCI), 0.25 μg/kg/min remifentanil, 100 μg fentanyl, and 35 mg rocuronium. General anesthesia was maintained with 2.7–4.0 μg/mL propofol (TCI) and 0.15–0.25 μg/kg/min remifentanil. For antiemesis, 4 mg ondansetron was given. The administration of 300 μg fentanyl and 1000 mg acetaminophen and rectus sheath block were done for multimodal analgesia.

The patient’s intraoperative body temperature was continuously measured by a nasopharyngeal thermometer, and it ranged from 36.1° to 36.5 ℃. The surgical data were as follows: surgical duration, 164 min; anesthesia time, 234 min; blood loss, a slight amount; urine output, 500 mL, total infusion volume, 1000 mL. The postoperative axillary body temperature measured periodically during the patient’s hospitalization ranged from 36.2° to 37.3 ℃, and perioperative cholinergic urticaria was not observed.

## Discussion

Careful body-temperature management is quite important during anesthesia in a patient with cholinergic urticaria. In general, anesthetics-induced thermoregulatory impairment can induce a patient’s hypothermia during a patient’s surgery [8], and an effort to warm a patient’s body is thus usually made by an anesthesiologist. However, passive warming should be avoided in a patient with cholinergic urticaria to prevent anaphylaxis following an increase in body temperature. In the present two patients, fluid warming and active cutaneous warming with a forced-air blanket were thus not provided, and the patients’ intraoperative body temperatures were managed by changing the operating theater temperature and by covering or removing a towel on their body surfaces. Excessive warming and a rapid increase in body temperature were thus avoided, which might have helped prevent the occurrence of intraoperative anaphylaxis due to cholinergic urticaria.

As shown by patient 2’s case, cholinergic urticaria is sometimes associated with cold-induced urticaria, and in some individuals with cholinergic urticaria, hives can be induced by colder living conditions with temperature differences [2, 7, 9]. Anesthesiologists should thus pay attention to the intraoperative risk of not only hyperthermia but also that of hypothermia to prevent anaphylaxis during surgery.

A passive warming test is performed when an individual is suspected of having cholinergic urticaria [10]. Details of the test are as follows: the subject stays in a hot (42 ℃) bath for 15 min after his or her body temperature increases ≥ 1 ℃ over the baseline body temperature, and a positive result is considered the appearance of pruritic pinpoint-sized wheals during the test or within ten minutes after the end of the test [10]. Considering the protocol of the passive warming test, the body temperature difference, e.g., an increase ≥ 1 ℃ over the baseline body temperature, may be an important risk factor for cholinergic urticaria. Indeed, the intraoperative body temperature differences were 0.2 ℃ in patient 1 and 0.4 ℃ in patient 2. It may be important to minimize a patient’s body temperature difference to prevent the occurrence of cholinergic urticaria during surgery.

Surgical stress produces various cytokines that increase the synthesis of prostaglandin E_2_ via cyclooxygenase-2 expression, which can induce hyperthermia [11]. Therefore, as described for patient 1, flurbiprofen, which is a non-selective cyclooxygenase-2 inhibitor, may be preferred in a patient with cholinergic urticaria. After surgery, a patient may experience various types of stress such as anxiety, pain, and nausea, and these stressors can also induce cholinergic urticaria [1]. In the two present cases, prophylactic antiemetics and multimodal analgesia were provided to minimize the patients’ postoperative stresses.

Each of the two present patients had no history of bronchial asthma, and their preoperative pulmonary function tests showed normal findings. However, the frequency of bronchial hyper-responsiveness in patients with cholinergic urticaria was noted to be 43% even when the patients had no history of bronchial asthma [12]. The patients’ intraoperative respiratory conditions were stable, but extra attention may be necessary for asthmatic bronchospasm when patients with a history of cholinergic urticaria were intubated and extubated.

## Conclusions

In patients with cholinergic urticaria, careful anesthetic planning including body-temperature management is necessary to achieve successful anesthetic management.

## Data Availability

The datasets for the reported patient are available from the corresponding author on reasonable request.
